# Effects of Parent-Delivered Traditional Thai Massage on Gait and Heart Rate Variability in Children with Autism: A Randomized Controlled Trial

**DOI:** 10.1089/jicm.2023.0338

**Published:** 2024-03-15

**Authors:** Hui Ruan, Wichai Eungpinichpong, Hua Wu, Chanada Aonsri

**Affiliations:** ^1^ Graduate School, Khon Kaen University, Khon Kaen, Thailand.; ^2^ Physical Education, Hainan Normal University, Haikou, China.; ^3^ BNOJHP Research Center, PT Division of Physical Therapy, Associated Medical Sciences, Khon Kaen University, Khon Kaen, Thailand.; ^4^ Department of Special Education, Khon Kaen University Demonstration School, Khon Kaen University, Khon Kaen, Thailand.

**Keywords:** autism, complementary and alternative medicine, heart rate regulation, movement disorders

## Abstract

**Aim:**

To examine the effects of parent-delivered traditional Thai massage (TTM) intervention on heart rate variability (HRV) and gait in children with autism.

**Methods:**

This was a two-armed, randomized controlled trial conducted at the Haikou Special Education School in Haikou Province, China, between October 2021 and March 2022. A total of 48 children with autism, aged between 7 and 12 years, were selected from the school and randomly divided into either the parent-delivered TTM group or the control group (no intervention) in a 1:1 ratio. In addition to their regular daily school routines, the TTM group received 16 TTM interventions (twice a week), with each session lasting ∼50 min. HRV and gait parameters were measured at baseline, completion of the 8-week intervention, and 2 months follow-up.

**Results:**

The results of this study showed that the TTM intervention had a notable positive effect on HRV, with a significant reduction in low-frequency value (*p* = 0.001), and increased high-frequency value (*p* = 0.001), compared with the controls, and the advantages persisted during the follow-up period. However, only the stride length in the TTM group was significantly longer than that in the control group at the post-test (*p* = 0.039) and follow-up test (*p* = 0.043), while none of the other parameters of gait comparison showed statistical significance.

**Conclusions:**

Parent-delivered Thai massage increased HRV levels and stride length in comparison to the control group, and some effects of the intervention were maintained over the follow-up period.

Clinical Trials Registry Identifier ChiCTR2100051355; September 21, 2021.

## Introduction

Autism spectrum disorder (ASD) is a neurodevelopmental condition caused by abnormal brain development and is characterized by core deficits in social interaction and communication, as well as restricted repetitive behavior patterns.^1^ ASD is typically identified in early childhood and is often challenging to accommodate throughout a person's lifetime. As per the most recent data published by the Centers for Disease Control and Prevention in 2023, the occurrence of ASD has increased by more than four times over the past 20 years, reaching a rate of 1 in every 36 children. Globally, the incidence is also increasing, with recent studies reporting an higher prevalence of 1% in countries such as China and India.^2,3^ This disorder affects millions of individuals and their families, and providing the necessary accommodations can incur great costs.

Other symptoms of ASD include movement disorders, atypical reactions to sensory stimuli, anxiety, hyperactivity, and sleep disorders; the aforementioned symptoms are believed to be linked with autonomic nervous system (ANS) dysfunction.^4–6^ Furthermore, autonomic dysfunction is manifested in the poor social emotion recognition and social regulation.^7–9^ Individuals with ASD have trouble accepting changes in their environment or schedule, leading to rigid and ritualized behavior.^10^ Heart rate variability (HRV) quantifies the fluctuation in time intervals between consecutive heartbeats or the corresponding instantaneous heart rate and serves as a widely accepted and noninvasive method.^11^ There is no consensus regarding whether there exists a distinction in HRV between individuals with ASD and those without the condition. However, most studies indicated that individuals with ASD exhibit lower levels of HRV than those without ASD.^12–16^

The presence of movement disorders—including abnormal gait, clumsiness, and irregular movement symptoms—is an additional characteristic that supports the diagnosis of ASD.^1^ Postural^17,18^ and gait abnormalities^19–21^ appear as early as infancy^22^ and persist throughout childhood^23,24^ and into adulthood.^25^ Previous studies have shown that dyskinesia can lead to atypical movements^26^ and limited social interaction^27,28^ and is related to sensory integration impairment,^25^ leading to the development of the core symptoms of ASD,^28,29^ which could reduce quality of life.^30^

In fact, atypical sensory responses (over- or under-responsiveness to sensory stimuli) have been included as part of the diagnostic criteria for ASD^1^ and are observed in 90% of children with ASD. Research has shown that the development of emotions, social skills, and cognitive abilities of children with ASD largely depends on early tactile experiences.^31,32^ Therefore, various tactile therapies, with massage as the predominant approach, have recently been utilized as complementary therapies for supporting the development of children with ASD.^8,33^ Research findings indicate that massage can have positive effects on sensory abnormalities and behavioral challenges in children with ASD.^34,35^ It provides gentle and rhythmic sensory stimulation, which promotes relaxation and emotional regulation.

In addition, a massage can enhance parent–child bonding and emotional communication, improving social interaction abilities in children with ASD.^34,36^ However, recent reviews have suggested that available evidence is limited in terms of demonstrating the effectiveness of massage in attenuating symptoms of ASD. These reviews highlighted issues such as limited sample sizes in the included studies, reliance on subjective assessments as primary outcome measures, inadequate control of nonspecific effects, and insufficient efficacy calculations or long-term follow-up.^37,38^ In addition, massage therapy has not yet been recognized as an evidence-based practice for ASD.^39^

Traditional Thai massage (TTM) is a part of Thailand's traditional medicine with a long history and is known for its unique combination of acupressure, stretching, and energy work.^40^ It focuses on improving energy flow, relieving tension, and promoting overall well-being.^41^ Evidence has shown that Thai massage could offer immediate or short-term effects that can enhance HRV^42,43^ and some gait parameters in normal adults and patients.^44–47^ However, its effects on children with ASD are still unknown. This study aimed to investigate the effects of parent-delivered TTM intervention on HRV and gait in children with autism.

## Methods

### Study design and setting

In this randomized controlled trial (RCT), children with ASD in grades 1–6 were randomly assigned to parental TTM or control groups, respectively. Experimental assistants used an online Research randomizer^48^ to generate random numbers (in a 1:1 ratio) and concealed the random numbers in opaque envelopes. The trial was conducted at the Haikou Special Education School in Haikou Province, China, between October 2021 and March 2022. All children provided written informed consent from their parents or guardians before inclusion in the study. The preregistration research protocol adhered to the ethical standards of the Declaration of Helsinki and was approved by the Khon Kaen University Ethics Committee for Human Research (ethical approval number: HE642167).

### Participants

The authors calculated that the minimum sample size was 48 participants.^49^ Parents of the participants were recruited through bulletin boards and verbal requests in the school. Patients aged between 7 and 12 years with a clinical diagnosis of ASD and no history of receiving full-body massage therapy were included. The inclusion criteria for parents included were as follows: had at least 1 year of parenting experience and were able to effectively communicate with the massage therapists.

Children with contraindications to TTM (e.g., fever, fractures, and psychoactive medications) or those receiving other specialized interventions were not considered during the study period. Parents with participation rates of <80% were excluded from the study.

### Blinding

The class teacher did not know which children have attended TTM intervention. Parents were asked not to discuss the intervention with others to prevent contamination of the control group. The data collectors were not informed of the grouping. The data are renamed by the experimental assistants and converted to statistics.

### Intervention

In addition to their regular daily school routines, the TTM group received 16 TTM interventions, with each session lasting ∼50 min. These sessions were conducted twice a week over an 8-week period in the school's health care room. Contrastingly, the control group continued to follow their daily school routines.

Before the intervention, the TTM experts provided three training sessions to parents in the TTM group. Each session lasted for at least 1 h and continued until the parents mastered the techniques. The training covered TTM theory, practice principles, and massage techniques and procedures. Only parents certified by the TTM experts were allowed to provide TTM interventions for the children. The TTM interventions were subsequently checked onsite by the TTM experts.

The TTM procedure involved massaging using the thumbs and palms, specifically focusing on pressing and adding extra pressure until gentle muscle resistance was observed. The muscles may feel tight but should not cause pain, followed by a 5–10 sec rest period. The specific details of the massage procedure have been outlined in a previously published protocol.^49^ If the participants experienced any adverse reactions or decided to withdraw from the study, the intervention was discontinued.

### Outcome parameters and data collection

Data were collected at baseline, after the eight weeks intervention, and two-month follow-up assessments.

### Heart rate variability

A UBiomacpa V1 device (Biosense Creative, Seoul, Korea) was used to assess the activity of the ANS. It measures parameters such as low frequency (LF), high frequency (HF), the ratio between LF and HF (LF/HF), mean beats per minute (BPM), standard deviation of normal-to-normal intervals (SDNN), and root mean square of the successive differences (RMSSD). This noninvasive device analyzes cardiac pulse variability in the fingertips based on established guidelines. It can detect stress levels and identify ANS abnormalities.

The HRV test was conducted in a closed room at a temperature of ∼26°C. Before the test, the administrator ensured that the device was connected properly and input the sex and age of the children with ASD. In the test, the children sat in the correct position while their index fingers were connected to a pulse sensor for 2 min and 30 sec. After the test, the computer automatically generated a data report.

### Gait

Gait analysis was conducted utilizing a portable BTS G-Walk device (BTS Bioengineering Company). This device captured gait data at a frequency of 100 Hz and wirelessly transmitted it to a computer via Bluetooth. The gait parameters measured have shown high reliability in test–retest and intertrial assessments (intraclass correlation coefficient: 0.728–0.969 and 0.84–0.99, respectively).^50,51^ Gait assessments were carried out on a level surface with a minimum area of 10 × 10 m and required a wireless internet connection. The participants' heights were recorded, and an elastic band with adjustable settings was positioned around their waist. They stood briefly before walking along a marked 7-m path while receiving verbal cues, but no physical assistance, from teachers or parents.

### Statistical analysis

Data analysis was performed utilizing IBM SPSS software (version 25.0; IBM, Armonk, NJ, USA). Demographic data are reported as mean ± standard deviation (SD) for continuous variables and as percentages for categorical variables. The differences between the two groups were assessed using an independent *t*-test. A mixed experimental design was employed, consisting of 3 (pretest, post-test, and follow-up) × 2 (TTM group, control group) conditions. The time factor (pretest, post-test, and follow-up) was considered within the groups, and the inter-group factor was the TTM group (control group). Based on this design, a simple effects analysis was conducted to better understand the data. Indicators with significant main and interaction effects in the statistical analysis were subjected to pairwise comparisons using a simple effects analysis. The significance level was predetermined at 5%, and the effect size was indicated by partial *ŋ*^2^ (*ŋp*^2^) as small (0.01), medium (0.06), or large (0.14).

## Results

**FIG. 1.**
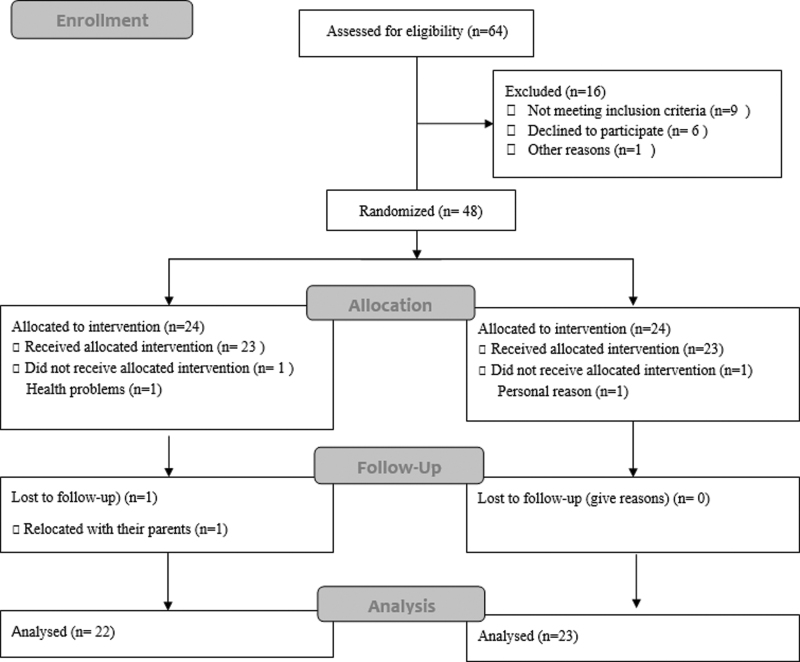
CONSORT 2010 flow diagram.

Among the 64 children with ASD screened, 48 were randomly assigned to either the TTM group (*n* = 24) or the control group (*n* = 24). During the study, there were two cases of attrition in the TTM group (one child withdrew due to illness and another relocated with their parents during the follow-up period). In the control group, one participant transferred to another school. Ultimately, data from 22 participants with ASD (21 males, 1 female) in the TTM group and 23 participants (22 males, 2 females) in the control group were included in the statistical analysis (Fig. 1). Table 1 presents the baseline characteristics of the study participants.

**Table 1.** Demographics of Participants

Variable	TTM group (*n* = 22)	Control group (*n* = 23)	*t*	*p*
Age (years)	10.00 ± 1.38	10.50 ± 1.33	1.25	0.22
Height (cm)	142.36 ± 13.15	145.57 ± 14.03	0.79	0.44
Weight (kg)	40.50 ± 15.62	41.72 ± 17.52	0.25	0.81
BMI (kg/m^2^)	19.24 ± 4.16	18.91 ± 5.11	−0.24	0.81
Child abnormal behavior (months)	27.14 ± 13.27	28.83 ± 11.91	0.45	0.66
Child confirmed age (months)	36.41 ± 12.72	38.04 ± 19.32	0.33	0.74

BMI, body mass index; TTM, traditional Thai massage.

### HRV parameters

The repeated-measures analysis of variance indicated significant main effects for both time and group, as well as a significant interaction effect observed between time and group, for the LF and LF/HF parameters. HF demonstrated significant main effects for both group and time, along with a significant interaction effect between time and group. The SDNN displayed significant main effects for time and a significant interaction effect between time and group. The RMSSD demonstrated a significant main effect of time. Furthermore, all these effects demonstrated effect sizes ranging from small to large (Table 2).

**Table 2.** Comparison of Heart Rate Variability among Pretest, Post-Test, and Follow-Up Between Groups

Outcome	Pretest	Post-test	Follow-up	Time			Group			Time × group		
Group	Mean ± SD	Mean ± SD	Mean ± SD	F	*p*	Partial* ŋ^2^*	F	*p*	Partial* ŋ^2^*	F	*p*	Partial* ŋ^2^*
LF												
TTM	7.74 ± 0.62	6.72 ± 0.61	7.22 ± 0.70	11.451	0.001^**^	0.21	13.456	0.001^**^	0.238	4.065	0.021^*^	0.086
CON	7.85 ± 0.84	7.60 ± 0.60	7.71 ± 0.68									
HF												
TTM	6.45 ± 0.91	7.24 ± 0.64	6.93 ± 0.52	2.457	0.092	0.054	16.954	0.001^**^	0.283	4.315	0.016^*^	0.091
CON	6.29 ± 0.86	6.12 ± 0.93	6.48 ± 0.86									
LF/HF												
TTM	1.24 ± 0.29	0.94 ± 0.16	1.05 ± 0.10	4.27	0.020^*^	0.169	24.16	<0.001^**^	0.36	4.64	0.015^*^	0.181
CON	1.27 ± 0.22	1.27 ± 0.25	1.22 ± 0.21									
SDNN												
TTM	46.88 ± 18.22	64.25 ± 24.36	55.53 ± 20.14	7.263	0.001^**^	0.144	2.092	0.155	0.046	3.154	0.048^*^	0.068
CON	48.21 ± 18.00	52.17 ± 20.17	45.25 ± 16.86									
RMSSD												
TTM	35.85 ± 12.61	49.23 ± 15.54	40.76 ± 13.54	9.812	0.001^**^	0.186	2.658	0.11	0.058	2.889	0.061	0.063
CON	34.13 ± 12.01	38.38 ± 12.42	37.01 ± 15.08									

A difference at the level of *p* < 0.05 is considered statistically significant.

CON, control; HF, high frequency; LF, low frequency; RMSSD, root mean square of successive differences between normal heartbeats; SD, standard deviation; SDNN, standard deviation of normal-to-normal intervals.

Further simple effect analyses were performed as follows:

Regarding the effect of time as an independent variable in the TTM group, the LF initially decreased and then increased over time. The post-test value was significantly lower than the baseline value (*p* = 0.001) and significantly higher than the follow-up test value (*p* = 0.009). The HF value significantly increased from baseline after the intervention (*p* = 0.01). The LF/HF ratio significantly increased from baseline (*p* = 0.001), and although it increased slightly during the follow-up period, it remained significantly lower than that at baseline (*p* = 0.026). The SDNN only showed a significant increase at post-test compared with baseline (*p* = 0.001). No significant changes were observed in these parameters over time within the control group.

**FIG. 2.**
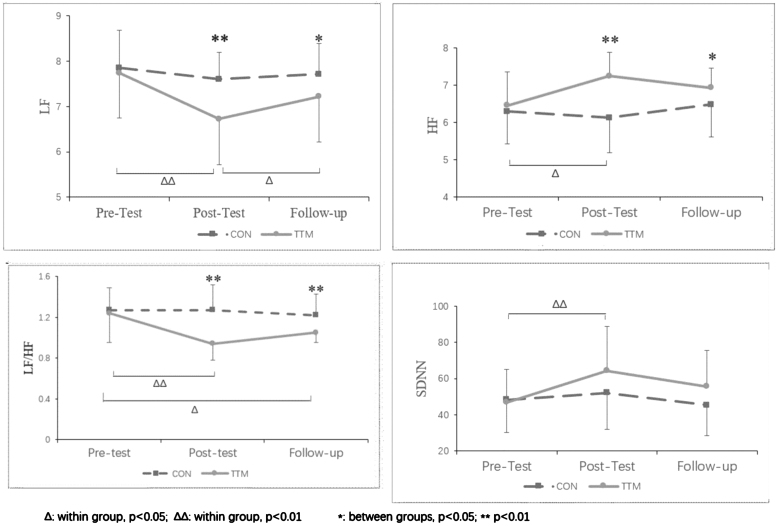
The simple effects analysis of the HRV parameters. HRV, heart rate variability.

Regarding the effect of group as an independent variable, significant differences were found between the two groups in the post-test and follow-up phases for LF, HF, and LF/HF values (Fig. 2).

### Gait parameter

**FIG. 3.**
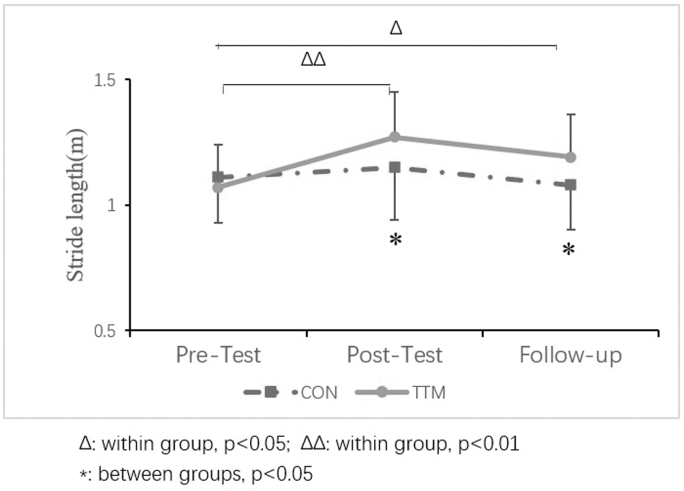
The simple effects analysis of the gait parameters.

The results showed that only the stride length had a statistically significant main effect for time [*F* (1,43) = 7.920, *p* < 0.001] and an interaction effect between time and group [*F* (1,43) = 4.303, *p* = 0.017] (Table 3). A simple effects analysis is presented in Figure 3. The effect of time as an independent variable indicated that the stride length of the TTM group increased after the intervention and was significantly longer than the baseline level (*p* = 0.001). Although the stride length decreased during the follow-up period, it was still longer than the baseline and was significant (*p* = 0.033). The changes in the control group at the three time points were not significant. With the effect of group as an independent variable, the stride length in the TTM group was significantly longer than that in the control group at the post-test (*p* = 0.039) and follow-up test (*p* = 0.043).

**Table 3.** Comparison of Gait Among Pretest, Post-Test, and Follow-Up Between Groups

Outcome	Pretest	Post-test	Follow-up	Time			Group			Time × group		
Group	Mean ± SD	Mean ± SD	Mean ± SD	F	*p*	Partial ŋ^2^	F	*p*	Partial ŋ^2^	F	*p*	Partial ŋ^2^
Stance phase (%)												
TTM	60.06 ± 1.94	60.48 ± 1.89	59.97 ± 1.71	1.915	0.154	0.043	4.047	0.051	0.086	0.014	0.986	0
CON	61.00 ± 2.51	61.48 ± 2.00	60.88 ± 1.62									
Swing phase (%)												
TTM	39.94 ± 1.94	39.52 ± 1.89	40.02 ± 1.71	1.915	0.153	0.043	4.047	0.051	0.086	0.014	0.986	0
CON	39.00 ± 2.51	38.52 ± 2.00	39.12 ± 1.62									
DS (%)												
TTM	9.66 ± 2.10	10.67 ± 1.35	10.21 ± 1.68									
CON	10.94 ± 2.27	11.10 ± 1.90	10.45 ± 1.58	2.42	0.095	0.053	2.357	0.132	0.052	1.689	0.191	0.038
SS (%)												
TTM	40.30 ± 1.85	39.77 ± 1.88	39.87 ± 1.75	1.036	0.359	0.024	3.904	0.055	0.083	0.95	0.391	0.022
CON	39.07 ± 2.26	38.85 ± 1.60	39.42 ± 1.67									
Cadence (steps per min)												
TTM	113.62 ± 13.79	112.05 ± 12.79	112.28 ± 12.90	0.817	0.445	0.019	0.074	0.787	0.002	0.546	0.581	0.013
CON	112.89 ± 12.29	113.30 ± 12.25	109.31 ± 12.22									
Speed (m/sec)												
TTM	1.00 ± 0.21	1.04 ± 0.14	1.08 ± 0.20	0.989	0.376	0.022	0.005	0.942	0	1.091	0.341	0.025
CON	1.02 ± 0.18	1.08 ± 0.20	1.02 ± 0.24									
Gait cycle duration (sec)												
TTM	1.13 ± 0.18	1.13 ± 0.17	1.14 ± 0.19	1.184	0.311	0.027	0.005	0.944	0	0.519	0.597	0.012
CON	1.12 ± 0.13	1.10 ± 0.14	1.17 ± 0.19									
Stride length (m)												
TTM	1.07 ± 0.17	1.27 ± 0.18	1.19 ± 0.17	7.92	0.001^*^	0.156	2.466	0.124	0.054	4.303	0.017^*^	0.091
CON	1.11 ± 0.18	1.15 ± 0.21	1.08 ± 0.18									

A difference at the level of *p* < 0.05 is considered statistically significant.

DS, first double support phase; SS, single support phase.

## Discussion

Owing to insufficient evidence on the effectiveness of massage therapy as an intervention for children with ASD, it is necessary to conduct research that delves deeper into the specific effects and mechanisms of massage therapy in individuals with ASD. This RCT aimed to examine the effects of a parent-provided TTM intervention on HRV and gait in children with ASD to provide improved guidance for clinical practice.

The findings of this study demonstrate that TTM decreases LF levels and increases HF values and that both SNDD and RMSSD increased after intervention in children with ASD, indicating stronger parasympathetic regulation and better autonomic regulation. The findings align with those of previous studies, showing that short-term massage can increase HRV.^52^ Autonomic features in individuals with ASD may linked to heightened reactivity and arousal in specific brain regions, such as the prefrontal cortex, insula, and anterior cingulate cortex, which are believed to play crucial roles in these mechanisms.^5^ Massage may increase HRV by modulating the ANS, and evidence has shown that massage increases vagus tone by stimulating tactile receptors (i.e., Pacinian corpuscles).^53,54^

Using functional magnetic resonance imaging techniques, massage therapy was found to have a modulating effect on areas of the brain associated with arousal and consciousness, such as the insula, posterior cingulate, anterior cingulate, inferior parietal cortices, and medial prefrontal cortices.^55^ However, massage therapy was also found to impact several brain regions involved in stress and emotion regulation, including the amygdala, anterior cingulate cortex, and hypothalamus.^54^ There is also evidence that massage can alter autonomic nervous function,^56^ increasing parasympathetic activity by lowering heart rate,^57^ and increase HRV.^52,58^ In addition to the effects of different brain regions, low HRV in individuals with ASD is associated with the neuroendocrine system.

Oxytocin is known to be associated with prosocial behavior, whereas the hypothalamic–pituitary–adrenal axis is associated with the stress response.^59^ Morhenn et al.^60^ found that massage could increase oxytocin levels and decrease adrenocorticotropic hormone, nitric oxide, and beta-endorphin levels, which may explain why social connections have been found to reduce morbidity and mortality rates. A study of seven children with ASD who received a maternal massage showed that during massage therapy, the concentration of oxytocin in both children and mothers was higher than during periods without massage.^61^ In one study, 24 adults with low-back pain experienced increased serotonin and dopamine levels after a 30-min massage intervention twice a week for a duration 5 weeks.^62^

The authors conducted studies on the effects of TTM after 8 weeks and during follow-up in children with autism. The results demonstrated that at both the post-test and follow-up time points, the stride length of the children in the TTM group was significantly longer than that of the children in the control group. This indicates that massage significantly improved the stride length and maintained its effect over time. Some people have suggested that children with ASD have difficulty with motor planning and coordination, which can affect their ability to initiate and sustain a fluid walking pattern.^63^ A quantitative review of the neuroimaging literature demonstrated notable differences in gray and white matter volumes specifically within the basal ganglia and cerebellum between the ASD and control groups. Neuroimaging studies have shown that these structures are not activated to the same extent in individuals with ASD when they complete motor tasks.

Abnormalities in the structure or function of the basal ganglia and/or cerebellum can affect the gait.^64^ In addition, some studies have suggested that children with ASD may have decreased muscle tone and postural stability, which can also contribute to a shorter stride length.^65^ TTM has been shown to improve gait by enhancing muscle function and flexibility, relieving muscle tension, improving proprioception, and enhancing coordination and balance.^66^ The authors also found that massaging and stretching relaxed the lower back muscles, quads, and hamstrings as well as the calf and plantar muscles, making walking more beneficial. One explanation for the absence of a significant finding on other gait parameters may be related to the variability of gait parameters,^67^ which is influenced by various factors, including individual differences as well as the effects of interventions and testing.

The strength of this RCT was the use of objective measures to explore the effects of massage on ASD. Furthermore, physiological indicators were used to speculate on the possible mechanisms underlying the effects of massage. This study has some limitations. The measurement of gait and HRV may be affected by many factors, including the heterogeneity of individuals with ASD; for example, gait may be affected by walking speed, and HRV may be affected by the degree of cooperation of children with ASD; however, the authors did try to control the distractions as much as possible during the test session.

## Conclusions

Parent-delivered Thai massage increased HRV levels and stride length compared with the control group, and some effects of the intervention were maintained over the follow-up period. This study offers a holistic and noninvasive approach to address motor and autonomic dysregulation symptoms commonly observed in autism. By involving parents and incorporating this intervention into comprehensive treatment plans, it can assist in the treatment and family-centered care of children with autism. Future studies should explore the mechanism by which Thai massage improves symptoms in children with ASD, including biochemical indicators and electroencephalogram parameters. In addition, the authors expect to explore long-term massage effects, such as 6 months or a year.
